# The Function of Occupational Activity for Health as Perceived by Chronically Ill People

**DOI:** 10.3390/ijerph19137837

**Published:** 2022-06-26

**Authors:** Katarzyna Mariańczyk, Wojciech Otrębski, Karolina Krzysztofik

**Affiliations:** Institute of Psychology, The John Paul II Catholic University of Lublin, 20-950 Lublin, Poland; otrebski@kul.pl (W.O.); karolina.krzysztofik@kul.pl (K.K.)

**Keywords:** chronic disease, employment, health, psychosocial problems

## Abstract

Existing studies confirm the benefits of employment for chronically ill persons’ health, but few studies so far have delved into how they themselves perceive employment in relation to their health. There is also a paucity of information about individual factors influencing the formation of their perceptions. This study sought to determine differences between chronically ill persons with and without jobs regarding their perceptions of the function of employment for the physical, mental and social dimensions of health, as well as how their occupational activity or inactivity moderates the associations between the perception of work as health beneficial or health adverse and selected individual characteristics, such as self-efficacy, acceptance of illness, actualisation of self, and psychosocial problems. The study involved 80 adults with chronic illnesses and was conducted using the following psychological tools: the *WH Scale*, the *Generalized Self-Efficacy Scale*, the *Acceptance of Illness Scale*, the *Actualization of Self Scale* and the *Psychosocial Problems of Persons with Chronic Illness Scale*. It has been found that the way in which chronically ill persons perceive the function of employment for health is unrelated to whether or not they have a job, and that occupational activity moderates associations between the sense of self-efficacy and the perception of work as benefitting health.

## 1. Introduction

The employment of adults has various dimensions and meanings. The decision whether or not to take a job is influenced by many factors, including one’s social situation, temperament and aspirations, openness to new experiences, and the need for self-development [1]. Activity in the professional sphere is not neutral to health, however. Its effects can be positive (such as a sense of development and fulfilment) or negative (occupational burnout, etc.) [2,3].

Employment is particularly challenging for people with chronic illnesses who frequently experience discrimination in the labour market [4], and so it provides many arguments against taking a job [5]. As a result, in many countries a strong emphasis has started to be placed on implementing various measures enabling chronically ill persons to start and stay in employment, such as the Polish campaign “Disease? I work anyway!” (2016–2019) launched by chronically ill persons determined to have personal and work lives like other people rather than rely on assistance from others or resort to sick leave or even disability pensions [6].

Alvani, Parvin Hosseini, and Alvani [2] report that unemployment is a strong stressor, especially for people who experience restrictions in daily functioning, activity, and social participation due to chronic illness, etc. According to research [7,8], anxiety over losing a job and financial stability dents self-esteem and increases the risk of depression. In patients with sclerosis multiplex, their lower mental wellbeing and reduced quality of life were found to be associated with the disease’s impact on their employment and income.

It was not until the 20th century that chronic diseases were noticed as a public health issue and a social challenge. According to the definition coined by the U.S. *National Center for Chronic Disease Prevention and Health Promotion* (NCCDPHP), chronic diseases are conditions that “last 1 year or more and require ongoing medical attention or limit activities of daily living or both”. They are also characterised by the permanent nature of the psychosomatic changes they entail. Among the chronic diseases that have the highest disability rates and which most frequently lead to death in the USA are cardiovascular conditions, tumours, and diabetes [9,10].

Chronic illness is a complex health issue because its biological, psychological, and physical aspects intertwine and interact with each other [11]. As a result, patients develop different strategies to cope with their condition, which has inspired the development of various theoretical frameworks and approaches to study them, i.e., biomedical, psychological (focusing on stress and the ways of coping with it), and self-regulatory. In most present-day studies, the biopsychosocial framework is used [12].

The clinical consequences of chronic diseases and the social experiences of those affected by them depend on how a disease develops and on its symptoms (e.g., the way a chronically ill person is treated by others is likely to be related to whether or not the symptoms are visible), as well as on the severity of psychosocial problems experienced [13]. As chronic illness is always accompanied by stress, discomfort, and pain, it requires adjusting life patterns to new daily challenges and creating strategies for protecting one’s mental and physical health. A chronically ill person may, for instance, feel forced to abandon or redefine the social roles they have fulfilled so far, to consider their spending patterns vis-à-vis financial stability, to seek retraining or modify their careers, and/or to accept the fact that their functioning may depend on assistance from family members or other people available to offer service [13,14,15]. A decision about whether to continue employment, to retire permanently, or to go out and find a job after a break thus becomes part of adapting oneself to living with a chronic disease [16].

Chronic diseases of various etiologies (oncological, rheumatic, neurological, etc.) are frequently diagnosed among working-age people fully enjoying family, social, and working lives [14]. A diagnosis of a chronic condition and the prospects of lifelong treatment give special meaning to work, comparable to that experienced by persons with disabilities [14,16]. Work becomes part of the daily struggle to have a normal life and continue its established rhythm; for many chronically ill persons, continuing treatment is a motivation for living [8,16,17].

Statistics show that around 30% of Europe’s working-age population suffers from chronic diseases [18]. In Poland, the economically inactive population exceeds 5.5 million adults, with an increasing share of people who do not work because of some chronic disease [19]. Studies show a high level of concern among chronically ill adults aged 50 or older that their condition may dramatically affect their careers, causing them to retire or start from scratch in a new profession. For many of them, the anxiety and uncertainty as to their health are strong stressors affecting their daily lives [20].

Health psychology and consequently public health science and health promotion science consistently argue that health should be understood comprehensively, i.e., not as an absence of disease, but rather as biopsychosocial wellbeing represented by a process and/or the exploitable potential of a person [21]. The definitional boundaries of health are steadily widening, and the need to take a broader perspective on health becomes especially distinct in the case of chronically ill persons who still have some health potential that can be strengthened and personal resources to overcome limitations.

Research reports show that the psychophysical and social wellbeing of humans is shaped by a variety of personal, social, and cultural factors, including occupational activity that according to Loisel [22] and Durand et al. [23] can have a positive effect on chronically ill persons’ health and their perception of it. It is also mentioned as an activity capable of preventing *work disability* [24]. This understanding of work accords with the multifaceted concept of adaptation to living with a chronic disease, which emphasises the current life context and psychosocial resources of chronically ill persons [25].

**Figure 1:**
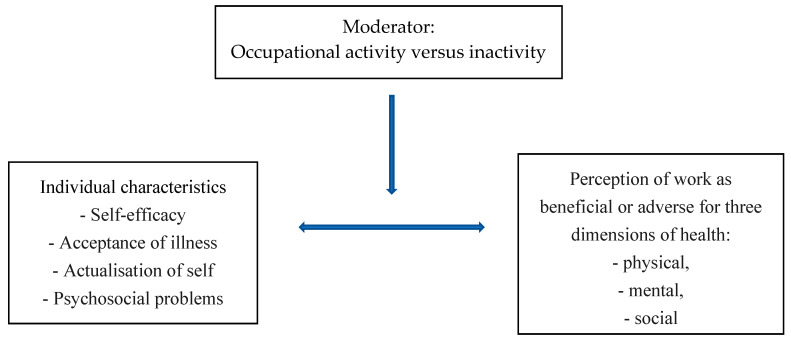
The theoretical design of the study.

Based on the existing studies and given that a number of questions have not yet been answered, this study was undertaken to determine in a group of chronically ill persons how having or not having a job moderates the association between a chronically ill person’s individual characteristics (self-efficacy, acceptance of illness, actualisation of self, and psychosocial problems) and their perception of work as health beneficial or health adverse (Figure 1).

The study addressed the following hypotheses:

**Hypothesis** **1** **(H1).** *Chronically ill persons who have a job view work as significantly more beneficial for mental and social health than those who are occupationally inactive.*

**Hypothesis** **2.1** **(H2.1).** *In chronically ill persons, perceived self-efficacy, acceptance of illness, and actualisation of self are significantly and positively associated with how they perceive the function of work for physical, mental, and social health.*

**Hypothesis** **2.2** **(H2.2).** *The intensity of psychosocial problems experienced by chronically ill persons is significantly and negatively associated with the perceived function of work for physical, mental, and social health.*

**Hypothesis** **3.1** **(H3.1).** *The individual characteristics of chronically ill persons (self-efficacy, acceptance of illness, actualisation of self, and psychosocial problems) condition their perception of work as health beneficial.*

**Hypothesis** **3.2** **(H3.2).** *The relationship between the aforementioned individual characteristics of a chronically ill person and their perception of the function of work for health is significantly moderated by the presence of occupational activity.*

## 2. Method

### 2.1. Study Design

The study was conducted with 80 persons with chronic illness at a mean age of M = 35.39 years (SD = 10.60). The sample was considered significative because of its clinical character and sufficiently representative to enable reliable inference about the psychosocial aspects of functioning.

The study participants were selected using random purposive sampling and the following inclusion criteria: being chronically ill at the time of the study and having or not having a job. Their contact data were acquired from databases, social media, internet forums, medical centres, and clinics. Information was collected during face-to-face interviews. All participants were informed that the study had a scientific purpose and that their participation was anonymous and voluntary.

### 2.2. Research Tools

The study involved the use of four questionnaires: the *Work–Health Scale* (W-H Scale); *Generalized Self-Efficacy Scale* (GSES); *Acceptance of Illness Scale* (AIS); *Actualisation-of-self Scale* (AS-5-R); and the *Psychosocial Problems of Chronically Ill Persons Scale* (PCH-R), all of which are briefly characterised below.

The *W-H Scale* by K. Mariańczyk (2019) is an experimental version of the tool developed to determine whether chronically ill persons perceive work as health beneficial or health adverse. It consists of 44 statements grouped into three sections, each one dealing with one dimension of health, i.e., physical (W-PH), mental (W-MH), and social (W-SH). Each item is assessed on a 5-point scale (where 1 = “I strongly disagree” and 5 = “I strongly agree”). A higher mean score on a health dimension means a more positive function of work for that dimension and vice versa. Cronbach’s alpha for the entire *W-H Scale* is 0.974 and for its respective sections is 0.832 (W-PH), 0.952 (W-MH), and 0.933 (W-SH).

The GSES, by Schwarzer and Jerusalem [26], is applied to determine a person’s general belief in their ability to cope with challenges and problems (known as self-efficacy). The Polish version of the scale was prepared by Juczyński in 2001. The GSES consists of 10 items rated on a 4-point scale, so the minimum and maximum scores are 10 to 40 points, respectively, with the perceived level of self-efficacy being higher the greater the score. The Polish scale has a Cronbach’s alpha of 0.85.

The *AIS* created by Felton et al. [27] measures adult patients’ acceptance of their illness and the degree to which they have learnt to live with it. The Polish variant was created by Juczyński [26]. The *AIS* includes 8 items rated on 5-point scales where 1 means “I strongly agree” and 5 means “I strongly disagree”. The minimum and maximum scores are 8 and 40 points, respectively, with acceptance being greater the higher the score. The AIS Cronbach’s alpha is 0.85 and the test–retest reliability 0.64, as is the case with its original version.

The AS-5-R by Witkowski, Wiącek, and Otrębski [28] assesses the level of actualisation of self, i.e., a process of self-development driven by purposeful and conscious actions. It consists of 16 bipolar scales for measuring 16 individual aspects of actualisation of self: setting life goals, being realistic, overcoming dichotomous thinking, attitude to time, sharing general human values, avoiding labelling of others, capacity for emotional contact with others, self-acceptance, focus on tasks rather than on defending oneself, inside locus of control, the occasional need for seclusion, the depth of emotional experiences, thinking independently rather than following other people’s opinions, a sense of humour, and creativity. Each aspect is measured on a 1-to-7 scale, where 1 means actualisation of self on this particular aspect and 7 denotes a lack of actualisation. The level of actualisation of self is determined based on the general score (AS-G) and the scores for four spheres: attitude to reality (AS-R), attitude to others (AS-P), self-perception (AS-Y), and self-expression (AS-E). The *AS-5-R* has a Cronbach’s alpha of 0.72 and uses sten norms.

The *PCH-R*, by Witkowski, Mariańczyk, Otrębski, and Wiącek [28], measures the severity of psychosocial problems experienced by chronically ill persons in four spheres: personality (PP-P), family (PP-F), society (PP-S), and occupational activity (PP-O). Each of the scale’s 60 items is rated on a 0–5 point scale. The general score (PP-G) and the scores for each of the spheres are calculated. The PCH-R has a Cronbach’s alpha of 0.95 and uses sten norms.

### 2.3. The Statistical Procedures

The SPSS program was used in the statistical analyses. The state of the analysed variables in the respective groups was described using the mean, standard deviation, and the distribution of frequency and percentages. The mean values in the respective responding groups were analysed using Student’s *t*-test or the Mann–Whitney U test. Pearson’s r coefficient was used to analyse correlations. In order to verify the theoretical model of moderation, linear regression was used with Andrew Hayes’ (2017) macro PROCESS version 3.4.1.

## 3. Results

### 3.1. Characteristic of the Study Sample

The study was conducted with 37 women and 43 men with chronic illness at a mean age of M = 35.39 years (SD = 10.60).

A total of 37.50% of them lived in rural areas, 40.10% in small- and medium-sized towns, and 20.00% in cities with populations above 100,000. In the group, 30.00% of participants had diabetes, 20.05% were diagnosed with cardiovascular diseases, 7.50% suffered from irritable bowel syndrome, and 5% were affected by multiple sclerosis, with the average disease duration being M = 8.32 years (SD = 8.56). Most participants (57.50%) were diagnosed less than 6 years prior to the study, 21.30% from 6 to 10 years, and 13.80% from 10 to 20 years. Only 7.50% of them had been ill longer than 20 years. Interestingly, all respondents who were employed indicated that they were assisted by others in their efforts to cope with their illness, compared with only two-thirds of those who did not work.

Most of those who had jobs (82.50%) were satisfied with this and 90.00% of them wanted to continue employment. More than one-third (37.50%) believed that employment was good for their health, 35.00% could not say whether it had any effect, and 27.50% thought that it impaired their health.

Among the occupationally inactive participants, 82.50% wanted to have a job, but only 52.50% actively sought one. Most persons in this group (70.00%) had worked before. Slightly less than half (42.50%) attributed their joblessness to their condition, and almost every second participant (47.50%) did not see their condition to be in any way connected to it; 27.50% considered themselves to be better off not having a job, and 25.00% were of the contrary opinion.

### 3.2. Perceptions of Work

The comparison of how participants who had jobs and those who did not perceived the function of work for the physical, mental, and social health showed that both groups tended to view its role as positive, with the tendency being the strongest for physical health (W-PH) in the first group (M = 3.91) and the weakest in the second group (M = 3.49). The two groups were not statistically significantly different from each other regarding their perception of the function of work for health dimensions (Table 1).

Participants’ answers on the W-H Scale were carefully analysed to find out more about how they perceived work in relation to physical, mental, and social health. The focus of the analysis was on statements with scores of 4 or 5 (indicating the participant’s belief in a positive relationship between work and health) and with scores 1 or 2 (revealing a negative perception of the role of work).

The occupationally active participants’ opinion that work is positively associated with health was based on their strong belief that employment makes a person feel useful and proves their ability to work. They also indicated that it helped organise daily activities and encouraged activity, was a chance to have a normal life, and contributed to building internal strength. Meanwhile, for the participants who did not have a job, the health benefits of work were mainly associated with the possibility of meeting other people and establishing positive relations with them and with a sense of security. It was also a clear indication that one continued to be a useful, employable, and important member of his or her community. The most important of all advantages of work, however, was that it offered financial independence.

With regard to the downsides of work, both groups found it to be physically and mentally straining and stressful. Only the occupationally inactive group indicated that a job made treatment of the disease more challenging, had a negative influence on physical health, did not contribute to increased activity, and did not help to think less about the disease. On the other hand, opinions that work impairs health, depletes energy, and makes attending to family matters and organising daily activities more difficult were only expressed by the occupationally active participants.

### 3.3. Factors Influencing Perceived Function of Work

In the group of the occupationally active participants, several statistically significant positive and negative correlations of weak or moderate strength were identified. The perception of work as benefitting physical health was related to acceptance of illness (*p* ≤ 0.05), while its benefits for mental health were associated with the general score for the actualisation of self (*p* ≤ 0.05) and self-perception (*p* ≤ 0.05). A positively perceived function of work for social health coincided with a lower intensity of psychosocial problems generally (*p* ≤ 0.01) and in each sphere—personality (*p* ≤ 0.05), social (*p* ≤ 0.05), family (*p* ≤ 0.05) and occupational (*p* ≤ 0.01)—as well as with better self-expression (*p* ≤ 0.05). Also, a greater sense of self-efficacy for these participants was found to be associated with their tendency to perceive work as benefitting all dimensions of health (W-PH *p* ≤ 0.05; W-MH *p* ≤ 0.01; W-SH *p* ≤ 0.05) (Table 2).

In the group of participants who did not have jobs, the intensity of psychosocial problems in the personality sphere was moderately strongly, negatively, and significantly related to the perceived function of work for physical (*p* ≤ 0.05), mental (*p* ≤ 0.05), and social (*p* ≤ 0.05) health. Additionally, the intensity of psychosocial problems generally and in the social sphere was moderately strongly, negatively, and significantly related to the perceived function of work for physical health (*p* ≤ 0.05; *p* ≤ 0.05). Thus, the greater the intensity of problems experienced by unemployed chronically ill persons in general and in the two spheres, the more likely they are to see work as adverse to health (Table 2).

### 3.4. Individual Characteristics Influencing Perceived Function of Work

The analysis revealed different configurations of relationships between personal characteristics and the perceived function of work for physical, mental, and social health between the occupationally active and inactive participants. Therefore, the extent to which personal characteristics contributed to work being perceived as beneficial for the dimensions of health was subsequently examined.

The occupationally active participants’ perception of work as supporting physical (β = 0.40; *p* = 0.011) and mental (β = 0.62; *p* < 0.001) health was positively determined by self-efficacy. However, participants’ perception of work as supporting social health was determined by actualisation of self in a dimension of self-expression (β = 0.48; *p* = 0.002) (Table 3).

The intensity of psychosocial problems in the personality sphere experienced by the occupationally inactive participants was found to be a negative determinant for the perceived function of work for physical (β = −0.42; *p* = 0.007) and mental (β = −0.45; *p* = 0.004) health. Also, the intensity of the problems in the social sphere negatively determined the perceived function of work for social health (β = −0.44; *p* = 0.005) (Table 4).

Lastly, the moderating effect of occupational activity and occupational inactivity on the relationships between the variables was analysed. The results presented below only concern the cases where the effect was statistically significant.

According to Table 5, the level of self-efficacy interacting with occupational activity/inactivity significantly contributes to work being perceived as beneficial for mental health. Therefore, occupational activity/occupational inactivity moderates the relationship between the sense of self-efficacy and the perception of work as beneficial for mental health.

In the occupationally active group, the positive relationship between the self-efficacy and the perception of work as beneficial for mental health was statistically significant (β = 2.01; *p* < 0.001). In the group of occupationally inactive participants, the same relationship was not significant (β = 0.66; *p* = 0.244). Therefore, the interaction of occupational activity with a sense of self-efficacy creates appropriate conditions for chronically ill persons to perceive work as beneficial for mental health.

## 4. Discussion

Three hypotheses were tested in the study. The findings did not confirm Hypothesis 1, stating that chronically ill persons who are occupationally active view work as more beneficial for mental and social health than those without jobs. The two groups were not different in how they perceived the function of work for each dimension of health. Nevertheless, the participants in both groups believed that work was beneficial for all three of them, as the groups’ mean scores on each dimension show. These results and the analysis of participants’ responses to the statements imply that chronically ill persons, whether employed or not, show some tendency to view work as beneficial for their health. However, the effect seems rather limited, which warrants a new study to determine why it is so. The study should analyse a wider range of the workplace determinants of occupational activity of chronically ill persons (including workplace accommodation, supportive vs. unsupportive climate at work, work–life balance, burnout, etc.) [4], as well as their life contexts and resources (e.g., personality) and the specific consequences of their diseases [8,29].

The results of this study also suggest a need for a closer examination of why chronically ill persons want to be occupationally active. An optimistic assumption that employment supports their biopsychosocial wellbeing seems risky, as it can divert attention from the problems they experience in the workplace, which can aggravate the symptoms of their disease and lead to more mental problems, increasing the risk of depression or occupational burnout [30,31]. The need to look at the problem under consideration from this angle is confirmed by the findings of a Dutch longitudinal study with 4820 chronically ill workers aged between 45 and 63 years. The authors of the study analysed the physical, mental, and emotional demands of their jobs, workplace autonomy, and social support available as factors that might influence the workers’ decisions to continue or terminate employment. They concluded that an improved match between the physical demands of the workers’ jobs and their capabilities could make them less likely to quit, whereas increasing psychosocial burden consolidated their decision to leave the employer, especially when the workplace environment offered them little support [32]. It seems, therefore, that for employment to noticeably benefit the health of chronically ill persons it must be accompanied by organisational, psychological, and other measures specifically addressing their individual needs [33], designed in line with solutions provided, for instance, in the ILO Code of Practice on Managing Disability *in the Workplace* [34].

Hypothesis 2 was tested separately for Hypotheses 2.1 and 2.2. Hypothesis 2.1 predicted that chronically ill persons’ self-efficacy, acceptance of illness, and actualisation of self would be significantly and positively associated with their perception of the function of work for the physical, mental, and social dimensions of health. Hypothesis 2.2 assumed a significant and negative relationship between the severity of psychosocial problems experienced by chronically ill persons and their perception of the function of work for the physical, mental, and social dimensions of health. Neither of the hypotheses was fully supported by the findings of the study. The belief that work was health beneficial, especially for its social dimension, was found to be associated with less-intense psychosocial problems in the personality and social spheres (in participants with and without a job) and in the family and occupational spheres (only in those who worked). The results support the message of the “Disease? I work anyway!” Polish campaign (http://www.pracujeznia.pl/ [accessed on 21 October 2021]) that employment can bring back normality into chronically ill persons’ lives, boost their self-confidence, and make them feel strong again, thus protecting them from the exacerbation of psychosocial problems. For persons confronted by chronic illness, employment is a confirmation that they are still able to oppose its consequences. This statement is confirmed by the results of our study, showing that the occupationally active persons’ stronger sense of self-efficacy was associated with their perceiving work as beneficial for all dimensions of health. It needs to be noted here that the sense of self-efficacy, which is partly related to continued occupational activity, is a key resource referred to in the context of health. Research shows that chronically ill persons with a stronger sense of self-efficacy are more likely to take better care of their health [35,36]. As the relationship only occurs among those who have jobs, it can be assumed that employment has a beneficial effect on the functioning of chronically ill persons.

Hypothesis 3 was tested separately for Hypotheses 3.1 and 3.2. Hypothesis 3.1 (self-efficacy, acceptance of illness, actualisation of self, and psychosocial problems condition perception of work as health beneficial in chronically ill persons) was partially confirmed. This implies that chronically ill persons perceive work to be positively associated with health for different reasons. In those who are occupationally active, the belief that work supports physical and mental health is positively determined by the sense of self-efficacy, with its perceived benefits for social health being also related to a high level of actualisation of self within self-expression. The results appear to be consistent with the findings of psychological studies on health behaviour factors, pointing to the sense of self-efficacy as one of the more important ones. Strengthening and developing the sense of self-efficacy of chronically ill persons contributes to their wellbeing [36,37,38], which is also related to the occupational activity. Active participation in a community of co-workers enables them to actualise their potential and express themselves [21].

Among the participants who were occupationally inactive, the perception of work as a factor contributing to physical and mental health was negatively determined by the intensity of psychosocial problems in the personality sphere and regarding social health by the intensity of the problems in the social sphere. These findings lead to the conclusion that for chronically ill persons to see a positive association between work and their health condition, circumstances favourable to this must occur, e.g., their psychosocial problems must not be so acute as to disqualify them from employment which contributes to better mental and social health. A combination of substantial psychosocial problems in the two spheres and the impacts of a chronic illness can become a significant or even insurmountable obstacle to engaging in occupational activity.

Hypothesis 3.2 (occupational activity significantly moderates relationships between the characteristics of chronically ill persons [self-efficacy, acceptance of illness, actualisation of self, and psychosocial problems] and the way they see the association between work and health) was only confirmed in part. A significant moderating effect of occupational activity was only determined for the relationship between self-efficacy and the function of work for mental health. This confirms, again, that a job and a sense of self-efficacy are necessary for chronically ill persons to see the benefits of work for mental health.

## 5. Conclusions

The study has shown that the chronically ill persons’ perception of the association between work and health depends on their self-efficacy and actualisation of self within self-expression, which are personal resources that continue to develop over a lifetime. If work is to benefit the persons’ health, the two resources should be enhanced both before and after they take up employment. The study has also found that for work to be more beneficial for the health of chronically ill persons, their self-efficacy and the level of self-actualisation need to improved, taking account of their individual life contexts defined by the disease and, above all, the course of the psychosocial problems it involves. The special importance of psychological counselling and social support for jobless persons with chronic conditions derives from their ability to attenuate the severity of psychosocial problems and restore belief in the benefits of work.

Having a job (or even being employed in the past) has been found to be associated with less severe psychosocial problems in the personality, social, and occupational spheres. Thus, occupational activity protects chronically ill persons from the exacerbation of psychosocial problems and naturally belongs to tertiary prevention. Tertiary prevention employs a wide range of measures to help people in need to improve physical fitness, overcome functional limitations and social exclusion, and develop interests and acquire skills they need to have active and independent lives. By delaying the progression of chronic diseases or disabilities, it makes it possible for people affected by them to retain or return to employment.

The awareness of the family members of chronically ill persons who are out of work that their close ones need support might prove helpful in convincing the latter that they need to go out and find work. Having a job is an opportunity to raise one’s quality of living and improve health potential, especially regarding its mental and social dimensions.

## Figures and Tables

**Table 1:** Perception of the function of work for health dimensions between occupationally active and inactive participants.

	Occupationally Active Participants *M (SD)*	Occupationally Inactive Participants *M (SD)*	*df*	*t*	*p*
*W-PH*	3.91 (0.71)	3.49 (0.85)	77	1.10	0.271
*W-MH*	3.82 (0.65)	3.56 (0.83)	77	1.55	0.124
*W-SH*	3.69 (0.73)	3.71 (0.97)	77	1.04	0.298

*M*—mean; *SD*—standard deviation; *t—*independent samples test statistic; *df*—the number of degrees of freedom; *p—*statistical significance; *W-PH*—the function of work for physical health; *W-MH*—the function of work for mental health; *W-SH*—the function of work for social health.

**Table 2:** Relationships between variables representing the individual characteristics of the occupationally active and inactive participants and the perceived function of work for health dimensions.

Personal Variables		Occupationally Active Participants			Occupationally Inactive Participants		
		Function of Work					
		*W-PH*	*W-MH*	*W-SH*	*W-PH*	*W-MH*	*W-SH*
*Psychosocial problems*	*PP-G*	−0.22	−0.25	−0.33 *	−0.036 *	−0.31	−0.30
	*PP-P*	−0.13	−0.19	−0.31 *	−0.37 *	−0.35 *	−0.33 *
	*PP-F*	−0.31 *	−0.26	−0.38 *	−0.27	−0.25	−0.27
	*PP-S*	−0.30	−0.24	−0.36 *	−0.31	−0.35 *	−0.35 *
	*PP-O*	−0.15	−0.17	−0.32 *	−0.23	−0.24	−0.25
acceptance *of illness*	*AIS*	0.34 *	0.25	0.21	0.12	0.14	0.06
*Self-efficacy*	*GSES*	0.39 *	0.58 **	0.35 *	0.16	0.16	0.14
*Actualisation of self*	*AS-G*	0.03	0.31 *	0.29	−0.01	0.08	0.04
	*AS-R*	−0.05	0.09	0.03	−0.09	−0.05	−0.09
	*AS-P*	−0.16	−0.01	−0.04	0.18	0.14	0.05
	*AS-Y*	0.08	0.37 *	−0.29	−0.06	−0.09	−0.17
	*AS-E*	0.18	0.30	0.40 *	0.05	0.14	0.12

*W-PH*—the function of work for physical health; *W-MH*—the function of work for mental health; *W-SH*—the function of work for social health; *PP-G—*general score for psychosocial problems; *PP-P—*psychosocial problems in the personality sphere; *PP-F*—psychosocial problems in the family sphere; *PP-S*—psychosocial problems in the social sphere; *PP-O*—psychosocial problems in the social sphere; *AI*S—acceptance of illness; *GSES*—self-efficacy; *AS-G*—general score for actualisation of self; *AS-R*—actualisation of self—the reality sphere; *AS-P*—actualisation of self—the social sphere; *AS-Y*—actualisation of self—self-perception; *AS-E*—self-actualisation—self-expression; ** *p* ≤ 0.01, * *p* ≤ 0.05.

**Table 3:** Regressions for the perceived function of work for health dimensions—the occupationally active group.

Explained Variable: *W-PH*				
*R^2^* = 0.16, *F* = 7.22, *p* = 0.011				
	*β*	*t*	*p*	95% CI
constant	13.97	2.50	0.017	2.65:25.30
*GSES*	0.40	2.68	0.011	0.11:0.84
**Explained variable: *W-MH***				
***R*^2^ = 0.38, *F* = 23.55, *p* < 0.001**				
	*β*	*t*	*p*	95% CI
constant	29.30	2.14	0.039	1.62:56.9
*GSES*	0.62	4.85	<0.001	1.23:2.99
**Explained variable: *W-SH***				
***R*^2^ = 0.23, *F* = 11.64, *p* = 0.002**				
	*β*	*t*	*p*	95% CI
constant	21.06	3.32	0.002	8.21:33.90
*AS-E*	0.48	3.41	0.002	0.38:1.51

*R***^2^**—model fit coefficient*; t—*test statistic*; β*—standardised regression coefficient; *SD*—standard deviation; *df*—the number of degrees of freedom; *p—*statistical significance; *W-PH*—the function of work for physical health; *W-MH*—the function of work for mental health; *W-SH*—the function of work for social health; G*SES*—self-efficacy; *AS-E*—actualisation of self in the self-expression sphere;

**Table 4:** Regressions for the perceived function of work for health dimensions—the occupationally inactive group.

Explained Variable: *W-PH*				
*R^2^* = 0.18, *F* = 8.08, *p* = 0.007				
	*β*	*t*	*p*	95% CI
constant	32.08	17.54	<0.001	28.37:35.79
*PP-P*	−0.42	−2.84	0.007	−0.25:−0.04
**Explained variable: *W-MH***				
***R*^2^ = 0.18, *F* = 9.36, *p* = 0.004**				
	*β*	*t*	*p*	95% CI
constant	104.00	17.00	<0.001	91.59:116.40
*PP-P*	−0.45	−3.06	0.004	−0.89:−0.18
**Explained variable: *W-SH***				
***R*^2^ = 0.19, *F* = 8.76, *p* = 0.005**				
	*β*	*t*	*p*	95% CI
constant	47.76	15.82	<0.00	41.64:53.88
*PP-S*	−0.44	−0.96	0.005	−0.50:−0.05

*R*^2^—model fit coefficient*; t—*test statistic; *β*—standardised regression coefficient; *SD—*standard deviation; *df*—the number of degrees of freedom; *p—*statistical significance; *PP-P*—psychosocial problems in the personality sphere; *PP-S*—psychosocial problems in the social sphere.

**Table 5:** Occupational activity as a moderator of relationships between self-efficacy and the perceived function of work for mental health.

Explained Variable: The Perceived Function of Work for Mental Health R^2^ = 0.17 *F* (3.76) = 19.46 *p* = 0.000					
	*β*	*SD*	*t*	*p*	95%CI
Constant	68.67	19.55	3.86	0.000	33.73:109.57
Self-efficacy	0.66	0.62	1.17	0.244	−0.64:1.82
Occupational activity vs. inactivity	−35.58	22.45	−1.75	0.083	−82.14:5.18
Self-efficacy × occupational activity and inactivity	1.35	0.70	2.12	0.036	0.06:2.78

*β*—standardised coefficient; *SD*—standard division; *t—*test statistic; *df*—the number of degrees of freedom; *p—*statistical significance.

## Data Availability

Data confirming the obtained and presented results are available from the authors of the article. If necessary, please contact the authors by email.
